# Lifetime stressor exposure, executive functioning, and internalizing symptoms during emerging adulthood

**DOI:** 10.1038/s41598-026-44738-4

**Published:** 2026-03-31

**Authors:** Liam Wright, Gloria Rebello, Dillon T. Browne, George M. Slavich, Mark Wade

**Affiliations:** 1 University of Toronto, Toronto, Canada; 2 University of Waterloo, Waterloo, Canada; 3 University of California, Los Angeles, USA

**Keywords:** Childhood adversity, Adulthood adversity, Stress, Executive function, Internalizing symptoms, Emerging adulthood, Diseases, Health care, Psychology, Psychology, Risk factors

## Abstract

Internalizing symptoms such as depression and anxiety rise dramatically during emerging adulthood. Although both childhood and adulthood adversity are associated with internalizing symptoms during this period, the underlying transdiagnostic processes connecting adversity to internalizing symptoms are unclear. To investigate this, we examined how childhood and adulthood adversity are related to internalizing symptoms during emerging adulthood via individual differences in executive functioning. In a cross-sectional study of 203 participants aged 18–24 years (*M*_age_=20.36, *SD*_age_=1.73, 66.01% women/transwomen, 28.08% White–European/North American), lifetime stressor exposure was indexed using the Stress and Adversity Inventory, internalizing symptoms were measured using the Kessler Psychological Distress Scale, and executive functioning was measured as a latent factor indicated by multiple performance-based measures. Analyses were conducted using regression and path analyses. In separate models, both childhood and adulthood adversity exposure predicted greater internalizing symptoms, but when entered as competing predictors in the same model, only adulthood adversity continued to predict psychopathology. No evidence for an indirect association involving executive functioning was detected, although higher childhood adversity was associated with *better* executive functioning. These results highlight differential associations between adversity timing, internalizing symptoms, and executive functioning during emerging adulthood, with minimal evidence of executive functioning as an indirect path linking adversity to internalizing symptoms.

**Supplementary Information:**

The online version contains supplementary material available at 10.1038/s41598-026-44738-4.

## Introduction

### Mental health during emerging adulthood

Depression and anxiety are leading causes of psychiatric morbidity worldwide, with recent reports from the Global Burden of Disease Report showing that the burden of both depression and anxiety continues to rise globally^1^. This burden is associated with increased costs to society, with projections as high as 12 billion days of lost productivity per year that translate into an estimated yearly cost of $925 billion USD^2^. Emerging adulthood is an especially unique period of social and cognitive change^3^ that is associated with elevated risk of internalizing problems such as depression and anxiety^4^. Indeed, results from the 2016 National Survey on Drug Use and Health demonstrate that emerging adults (18–25 years) were the group that experienced the highest level of past year major depressive episodes, with a reported prevalence of 10.9% compared to 7.4% in those aged 26–49 years, and 4.8% in those aged 50 and older^5^.

Despite higher prevalence rates among emerging adults, mental health service utilization is lower in this demographic^6^, which may further contribute to the individual and societal burden associated with depression and anxiety at this stage of development. Emerging adulthood therefore represents a crucial period to understand the antecedents and indirect pathways contributing to internalizing problems^4^. In the present study, we sought to investigate how experiences of adversity in both childhood and early adulthood are associated with internalizing difficulties during emerging adulthood, and to then examine executive functioning as a potential transdiagnostic process through which adversity is associated with internalizing problems.

### Childhood adversity versus adulthood adversity

One psychosocial factor that has been widely linked to the onset of depression and anxiety across the life course is childhood adversity. Population-based studies estimate that approximately one-quarter of all depression-related cases and one-third of anxiety-related cases are explained by childhood adversity^7^. In turn, preventing the negative consequences of childhood adversity may lead to reductions as large as one-in-four cases of depression at the population level^8^. Recent meta-analytic reports show that more than half of all individuals report having experienced some form of childhood adversity, and over one-third report having experienced two or more forms of adversity^9^. Moreover, childhood adversity portends an earlier age of onset of depression compared to non-maltreated individuals^10^, and has been indexed as being tied to more severe cases of both depression and anxiety in adulthood^11^. Emerging adulthood is therefore an especially important window to examine the effect of childhood adversity on internalizing problems, especially given the fact that childhood adversity tends to be associated with cases of internalizing problems that are more severe and treatment-resistant^10,11^.

Compared to the well-established literature linking childhood adversity to poor physical and mental health outcomes across the life course^12^, much less research has focused on the relationship between adulthood adversity and physical or mental health. Despite recognition of the importance of measuring adversity across the lifespan, most research focuses on adversity during childhood and its association with adult health outcomes. Cumulative lifespan stress models are relatively sparse by comparison, largely due to the fact that few measures exist that allow researchers to comprehensively index lifetime adversity in a cost-effective and efficient manner. This issue has been partially addressed by the development of the Stress and Adversity Inventory (STRAIN), which allows for the measurement of adversity across the lifespan and has revealed important associations with poorer physical, cognitive, and mental health outcomes from childhood to adulthood^13–16^. Moreover, only a handful of studies have contrasted associations between childhood adversity versus adulthood adversity and mental health outcomes. In one example, Stern and Thayer^17^ reported that both childhood and adulthood adversity were significantly associated with depression outcomes in young adulthood when indexed separately, but had a cumulative effect on depression when both were combined and modeled as lifetime exposure to adversity. Likewise, Zou et al.^18^ reported that both childhood and adulthood adversity were uniquely associated with the onset of cardiovascular disease, and that associations between childhood and adulthood adversity with cardiovascular disease were significantly mediated by depression symptoms. While both papers highlight that childhood and adulthood adversity may be independently associated with depression symptoms, competing effects of childhood and adulthood adversity modeled together remain poorly understood. Thus, the first goal of the present study was to understand associations between childhood and adulthood adversity, modelled both separately and together, with internalizing symptoms in emerging adulthood. The second goal was to test whether associations between childhood and adulthood adversity with internalizing problems operated indirectly through executive functioning, given its role as a transdiagnostic predictor of psychopathology^19^.

### Executive functioning as an indirect pathway to psychopathology

Despite clear associations between adversity and internalizing symptoms, transdiagnostic processes connecting adversity to mental health risk remain poorly understood^20^. However, executive functioning difficulties have been shown to be associated with increased risk of a range of mental health problems, leading many to conclude that this is a transdiagnostic risk for broad-spectrum psychopathology (i.e., the “*p* factor”^21^. Executive functioning describes a range of cognitive processes that are involved in goal-directed action, such as working memory, cognitive flexibility, response inhibition, and attentional control^22,23^. Recent meta-analytic work suggests that executive functioning challenges serve as a transdiagnostic factor that underlies or maintains risk for internalizing disorders across various age groups^19,24,25^.

In addition to the general association between executive functioning and psychopathology, there is also compelling meta-analytic data showing an association between childhood adversity and executive functioning in both youth^26,27^ and adults^28^. Notably, however, Lund et al.^28^ reported a paucity of studies examining associations between childhood adversity and executive functioning outcomes among emerging adults, with only three studies focused on individuals aged 18–24 years^29–31^ and only two of those using performance-based measures of executive functioning^29,30^. This is important as self-reports of executive functioning do not measure the same underlying construct as performance-based tasks^32^. Performance-based measures of executive functioning provide a better indication of cognitive processing efficiency and are therefore better markers of functioning in settings such as school and work. Self-report measures may also be prone to rater bias, and when coupled with the self-report nature of exposure to childhood adversity, are more susceptible to shared reporter bias that can artificially inflate associations between constructs. Thus, the current study used a direct performance-based measure of executive functioning.

In addition to robust evidence linking childhood adversity to executive functioning, as well as executive functioning to psychopathology, there is emerging evidence showing that executive functioning difficulties partially account for associations between childhood adversity and internalizing problems in both youth^33^ and young adults (i.e., undergraduates)^34^. However, there is comparatively little research on the association between adulthood adversity and executive functioning, and very little research that has included both childhood and adulthood adversity in the same study. One very large recent study of over 500,000 individuals in the UK Biobank showed that both childhood and adulthood stress were associated with decreased executive functioning in both males and females^35^. However, these participants were all middle-aged, with no representation from emerging adults. In another recent study, this time focused on participants aged 18–24 years, Shields and Hunter^36^ investigated time-dependent associations of adversity on cognition, showing that a greater degree of stressor mismatch between early and recent-life adversity was associated with *improved* response inhibition scores. A mismatch between early and recent stress may reflect uncertainty or instability in the environment that is associated with adaptations to that uncertainty, consistent with models such as the Hidden Talents Approach, which proposes that adversity may engender cognitive advantages that promote adaptation in unpredictable environments^37^. Still, no study has examined associations between both childhood and adulthood adversity in relation to executive functioning, and tested whether executive functioning accounts for associations between adversity and internalizing symptoms in emerging adulthood. Such a model is critical to understand whether the timing of adversity matters in the development of internalizing problems, and whether the indirect pathways linking adversity exposure to internalizing problems differ based on this timing.

### Present study

The present cross-sectional study aimed to examine whether self-reported childhood and adulthood adversity was associated with internalizing problems during emerging adulthood, and whether this effect operated indirectly through executive functioning. To do so, we recruited a sample of young adults who reported on a range of stressors during both childhood and adulthood. They also completed a questionnaire on their overall internalizing problems, and completed a battery of executive functioning tasks using a widely used and well-validated performance-based measure. All study-related aims and hypotheses were pre-registered following full data collection and data cleaning, but prior to any form of data analysis (10.17605/OSF.IO/4GNS5). Based on the research summarized above, we hypothesized that: (a) both childhood and adulthood adversity would both be associated with internalizing symptoms in emerging adulthood; (b) both childhood and adulthood adversity would be associated with lower executive functioning performance; and (c) associations between both childhood and adulthood adversity with internalizing symptoms would operate indirectly through executive functioning. We hypothesized stronger associations involving childhood adversity compared to adulthood adversity given the now well-developed literature that childhood is a period of heightened neuroplasticity during which the brain is highly malleable and responsive to experience^38^. Through this lens, it was hypothesized that adversity during childhood would be expected to have a more significant and lasting effect on the neurocognitive phenotypes underlying mental health risk.

## Method

### Participants

Participants were 209 young adults aged 18–24 years old. They were recruited from the area in and surrounding the University of Toronto using community-based advertisements, and through the University of Waterloo’s SONA undergraduate recruitment system. Exclusion criteria for study participation included the inability to read instructions written in the English language and being outside of the target age range. Four participants were excluded listwise from the study due to not passing attention checks on the Stress and Adversity Inventory (STRAIN; see below), and two participants were excluded due to missing data on all available measures. The final analytic sample size was thus 203 participants (*M*_age_=20.36; *SD*_age_=1.73, 66.01% women/transwomen, 28.08% White–European/North American). All procedures in the study were approved by both the University of Toronto Research Ethics Board (REB protocol #40683) and the University of Waterloo Research Ethics Board (REB protocol #43320). All study methods were performed in accordance with relevant institutional guidelines and regulations, and informed consent was obtained from all participants prior to study commencement. Data was collected over a six-month period between October 2021 and April 2022. Further demographic data can be found in Table 1.

Participants completed all study-related tasks and questionnaires virtually. Participants were sent independent links to a demographics survey and the self-report and performance-based measures (described below) and were instructed to complete the battery of assessments in this order. Participants completed all measures unsupervised on their personal devices, providing email confirmation after task completion. Following completion, data were checked to ensure all materials had been completed and participants were compensated via a $40.00 Amazon gift card and/or course credit.

**Table 1:** Sample characteristics (*N* = 203).

	*N* (%)	M (SD)	Range		Missing data %
Age (years)		20.36 (1.73)	18–24		2.46%
Gender					0.99%
Woman/transwoman	134 (66.01%)				
Man/transman	48 (23.65%)				
Genderqueer/gender non-conforming	3 (1.5%)				
Gender non-binary	3 (1.48%)				
Gender fluid	1 (0.49%)				
Prefer to self-define	2 (0.99%)				
Prefer not to answer	6 (2.96%)				
Man/transman, gender fluid	1 (0.49%)				
Woman/transwoman, genderqueer/gender non-conforming	1 (0.49%)				
Genderqueer/gender non-conforming, gender non-binary	2 (0.99%)				
Ethnicity					1.97%
Asian – East	43 (21.18%)				
Asian – South	57 (28.08%)				
Asian – South East	2 (0.99%)				
Black – African	4 (1.97%)				
Black – Caribbean	4 (1.97%)				
Black – North American	5 (2.46%)				
First Nations	2 (0.99%)				
Indian – Caribbean	4 (1.97%)				
Latin American	3 (1.48%)				
Middle Eastern	7 (3.45%)				
White – European/North American	57 (28.08%)				
Mixed heritage	8 (3.94%)				
Other(s)	2 (0.99%)				
Do not know	1 (0.49%)				
Years of education		14.62 (2.03)	3–18		0.99%
Monthly household income (Total responded)					3.94%
$0–2,000 CAD	30 (14.78%)				
$2–4,000 CAD	33 (16.26%)				
$4–6,000 CAD	34 (16.75%)				
$6–8,000 CAD	23 (11.33%)				
$8–10,000 CAD	20 (9.85%)				
$10,000 + CAD	55 (27.09%)				
Internalizing symptoms (K10)		14.67 (8.72)	0–39		0.98–5.42%†
Childhood adversity		5.48 (5.32)	0–26		0.00%
Adulthood adversity		6.93 (6.43)	0–33		0.00%
Recent-life adversity		2.59 (2.73)	0–14		0.00%
Childhood adversity severity		14.24 (14.05)	0–72		0.00%
Adulthood adversity severity		15.15 (15.11)	0–75		0.00%
Recent-life adversity severity		9.24 (10.64)	0–51		0.00%
SWM (rescaled)‡		28.13 (8.12)	0–35		5.42%
OTS		11.05 (3.04)	0–15		6.40%
RVPA		0.90 (0.07)	0.70–1.00		6.40%
IED (rescaled)¶		82.22 (28.46)	0–105		5.42%

† Missing data reported on the K10 reflects a range of missing responses at the item-level across the 10 items summed to calculate a total score.

‡ This is the rescaled mean for SWM. The original mean, standard deviation, and range were: M = 6.87; SD = 8.12; Range = 0–35.

¶ This is the rescaled mean for IED. The original mean, standard deviation, and range were: M = 26.78; SD = 28.46; Range = 4–109.

### Measures

#### Childhood adversity and adulthood adversity

Exposure to both childhood and adulthood adversity were indexed via participant self-report on the Stress and Adversity Inventory (STRAIN). The STRAIN assesses 55 different stressors over 220 questions that gather information on stressor severity, frequency, exposure timing, and duration (see https://www.strainsetup.com). From the raw item-level response data, 115 lifetime stressor summary scores are generated, with childhood and adulthood adversity being two of these scores. Childhood adversity is restricted to adversities experienced up to 18 years old, whereas adulthood adversity is restricted to adversities experienced from 18 years old onward. Given our sample age was limited to participants between 18 and 24 years, experiences of adulthood adversity were isolated to this period. Childhood and adulthood adversity exposures were pulled from the same pool of items within the STRAIN, but are filtered based on whether they are reported to have occurred before (i.e., childhood adversity) or after (i.e., adulthood adversity) age 18 years. In this way, the STRAIN indexes exposures that may be common across developmental timepoints, but also those that may be specific to a given developmental period. Our primary analyses operationalized childhood adversity as the total count of participant-endorsed stressors experienced before 18 years, whereas adulthood adversity was operationalized as the total count of participant-endorsed stressors between 18 and 24 years. Exploratory analyses also examined the severity of these exposures, with severity operationalized as subjective ratings of how severe participants believed the stressors to be during each period on a scale ranging from 1 to 5 (most severe). The distribution of our childhood adversity and adulthood adversity variables (both total count and severity) were non-normal and right-skewed, characterized by a concentration of values near the lower end of the scale and a long right tail.

In exploratory analyses, we also examined associations involving recent-life adversity, which was operationalized as the total count of stressors experienced within the last six months. As with childhood adversity and adulthood adversity, recent-life adversity severity was also explored. Additionally, while not part of our original pre-registration, in response to a reviewer comment we ran a cumulative stress model that combined both childhood and adulthood adversity into single index of “cumulative adversity” across the lifespan. The STRAIN has been well-validated, has strong psychometric properties^13^, and has been widely used to measure the effects of stressors on psychological outcomes such as anxiety, depression, and well-being^14–16^.

#### Internalizing symptoms

Internalizing symptoms were measured using the Kessler Psychological Distress Scale (K10). The K10 is a widely used and well-validated measure of unidimensional non-specific psychological distress symptoms related to depression and anxiety^39^. The K10 is widely used as a screening tool for internalizing symptoms and has been shown to have strong psychometric properties^39^. In the current study, the internal consistency was excellent (Cronbach’s Alpha = 0.91). The distribution of internalizing symptoms was roughly normal, with no evidence of bimodality. To characterize clinical risk in this sample, we followed recommendations by Slade et al.^40^, for non-clinical samples within the general population. Specifically, 39.90% of participants met a clinical cutoff of having K10 scores higher than 15 (“moderate” levels), 21.18% had scores higher than 21 (“high” levels), and 6.40% had scores higher than 29 (“very high” levels).

#### Executive functioning

Executive functioning was assessed across a series of tasks on the Cambridge Neuropsychological Test Automated Battery (CANTAB; http://www.cantab.com), a direct performance-based measure of executive functioning that demonstrates strong psychometric properties across age groups^41^. We included tasks assessing a range of executive functions: Spatial Working Memory (SWM; working memory), One-Touch Stockings of Cambridge (OTS; planning/decision-making), Rapid Visual Information Processing (RVP; sustained attention), and the Intra-Extra Dimensional Set Shift (IED; rule acquisition and attention shifting/flexibility). A single outcome from each of these tasks was selected in order to index performance: *SWM between errors* (i.e. the number of times a participant incorrectly revisits a box in which a token has been previously found), *OTS problems solved on first choice* (i.e., the number of trials where the participant chose the correct answer on their first attempt), *RVP A-prime* (i.e., sensitivity to target sequences regardless of participant response tendencies/how well the participant performs at detecting target sequences), and *IED total errors adjusted* (i.e., the total number of times the subject chose an incorrect stimulus/a measure of participant response efficiency). These metrics were used as indicators of a latent executive functioning factor capturing overall executive functioning (described below).

### Analyses

In descriptive analyses, bivariate correlations were examined between all study variables. In primary analyses, a latent executive functioning factor was estimated using confirmatory factor analysis (CFA) with each of the four CANTAB task outcomes as indicators. SWM and IED scores (higher scores reflecting more errors) were rescaled such that higher represented better executive functioning performance (fewer errors) to ensure all indicators were scaled in the same direction. Following a demonstration of adequate measurement model fit for the executive functioning factor, we tested three different structural equation models in which childhood adversity (Model 1), adulthood adversity (Model 2), and both childhood and adulthood adversity (Model 3) predicted internalizing symptoms through the latent executive functioning factor. We report total effects (c paths), direct effects (c’ paths), and indirect effects (a*b paths) as well as paths comprising indirect effects (i.e., stressors predicting executive functioning [a paths]; executive functioning predicting internalizing symptoms [b paths]). Model fit was evaluated based on the following criteria: A non-significant χ^2^ test, Comparative Fit Index (CFI ≥ 0.90), Tucker-Lewis Index (TLI ≥ 0.90), Root Mean Square Error of Approximation (RMSEA ≤ 0.06), and Standardized Root Mean Square Residuals (SRMR ≤ 0.08)^42,43^.

Three exploratory analyses were also conducted. First, all models reported above were replicated substituting the *total count* of stressors with *severity* scores. Second, we tested a *stress sensitization model* wherein the interaction between childhood and adulthood adversity was examined in relation to internalizing symptoms^44^. For these analyses, predictors were mean-centered to reduce multicollinearity among variables and aid in interpretation of the effects^45^. Finally, we tested a second stress-sensitization model with adulthood adversity substituted for recent-life adversity in the aforementioned interaction models.

In all models, age, ethnicity, gender, years of education, and monthly household income were entered as covariates per our pre-registered analysis plan. Ethnicity, gender, years of education, and monthly household income were dummy coded with the reference categories as White – European/North American, woman/transwoman, and monthly household income of >$10,000.00 CAD respectively. These reference categories were chosen based on the most frequent groups (see Table 1).

Descriptive statistics were conducted using SPSS version 29.01.0. Primary analyses were conducted using structural equation modelling in Mplus version 8.8 using a maximum likelihood estimator and 5,000 bootstrap samples to evaluate the significance of indirect effects^46^. Missing data was imputed using the missRanger package in R 2.6.2 for individual items and missing covariate data^47^. Outliers were set as values exceeding three times the interquartile range either below quartile one or above quartile three and were winsorized with replacement at the next highest value in the dataset^48^.

## Results

### Descriptive results

Descriptive results are reported in Table 1. Bivariate correlations between all study variables are reported in Table 2 (an expanded correlation table for all study variables is presented in Table S1). While purely descriptive, notable associations included significant positive correlations between childhood and adulthood adversity, significant positive correlations between both childhood and adulthood adversity with internalizing symptoms, and significant correlations between individual executive functioning task scores, though none of these were individually associated with childhood adversity, adulthood adversity, or internalizing symptoms. The significant inter-correlations between individual executive functioning measures justifies their inclusion as joint indicators of an overarching executive functioning latent factor.

**Table 2:** Bivariate associations between study variables.

	1. Age	2. Ethnicity (Reference Category: White – European/ North American)	3. Gender (Reference Category: Women & Transwoman)	4. Years of education	5. Monthly Household Income (Reference Category: $10,000+ CAD)	6. Internalizing Symptoms (K10)	7. Childhood Adversity	8. Adulthood Adversity	9. SWM (Rescaled)	10. OTS	11. RVPA	12. IED (Rescaled)
1.	﻿–											
2.	− 0.02	–﻿										
3.	0.25***	− 0.02	﻿–									
4.	0.61***	− 0.11	0.18*	﻿–								
5.	0.09	0.00	− 0.06	0.07	﻿–							
6.	− 0.12	− 0.09	− 16*	− 0.14*	− 0.04	﻿–						
7.	﻿− 0.26***	﻿− 0.15*	− 0.12	− 0.16*	− 0.06	0.30***	﻿–					
8.	0.02	− 0.10	− 0.00	− 0.03	− 0.07	0.42***	0.43***	﻿–				
9.	0.24***	− 0.06	0.01	0.15*	0.01	0.00	− 0.07	﻿− 0.06	﻿–			
10.	0.15*	**− ﻿** 0.17*	− 0.02	0.19**	0.02	0.07	0.13	0.04	0.32***	﻿–		
11.	0.23**	− 0.16*	0.01	0.29***	− 0.05	0.04	0.13	0.06	0.42***	0.53***	﻿–	
12.	0.14	− 0.04	0.01	0.11	0.06	− 0.08	0.05	− 0.03	0.28***	0.38***	0.35***	﻿–

***p ≤.001. **p ≤.01. *p ≤.05 reflect two-tailed *p*-value. Bolded coefficients are significant at *p* <.05 (two-tailed).

### Measurement model

The measurement model for executive functioning is presented in Figure S1. Model fit was good: χ^2^ = 2.48 (df = 2; *p* =.29), RMSEA = 0.03 (90% CI 0.00, 0.15; *p* =.46), CFI = 1.00, TLI = 0.99, SRMR = 0.02 and all indicator loadings were significant (*p* <.05). Thus, a coherent factor representing overall EF was established based on the four CANTAB task indicators.

### Structural models

**Fig. 1:**
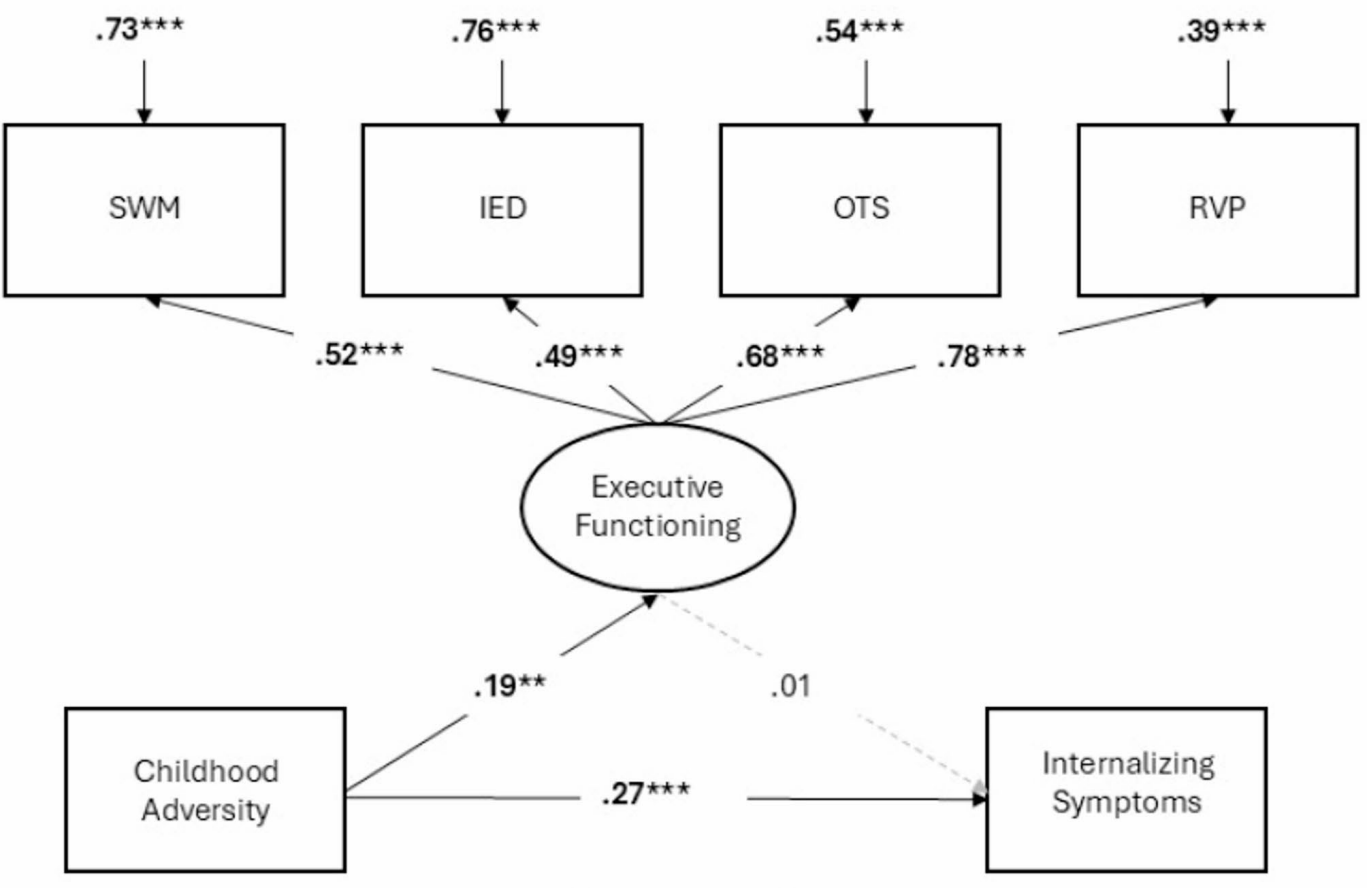
Results from structural equation model linking childhood adversity to internalizing symptoms through executive functioning. Coefficients are standardized beta coefficients, with significant paths presented as solid lines with bolded coefficients, while gray/hashed lines are non-significant. *** *p* ≤.001, ** *p* ≤.01, * *p* ≤.05 two-tailed test).

**Fig. 2:**
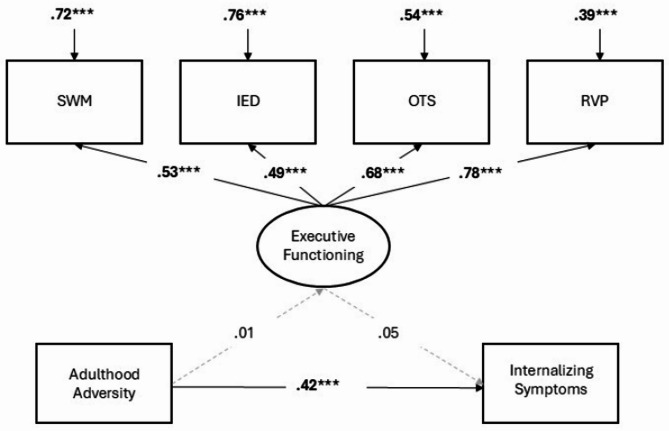
Results from structural equation model linking adulthood adversity to internalizing symptoms through executive functioning. Coefficients are standardized beta coefficients, with significant paths presented as solid lines with bolded coefficients, while gray/hashed lines are non-significant. *** *p* ≤.001, ** *p* ≤.01, * *p* ≤.05 two-tailed test).

**Fig. 3:**
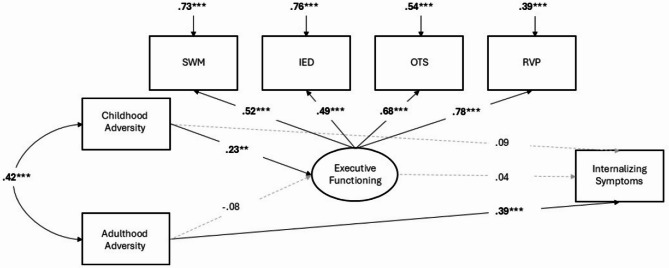
Results from structural equation model linking Childhood Adversity and Adulthood adversity as simultaneously competing predictors to internalizing symptoms through executive functioning. Coefficients are standardized beta coefficients, with significant paths presented as solid lines with bolded coefficients, while gray/hashed lines are non-significant. *** *p* ≤.001, ** *p* ≤.01, * *p* ≤.05 two-tailed test).

Model fit statistics were good for all three structural models (see Table 3). Results for all three models are reported in Table 3. The total effect of adversity on internalizing symptoms was significant in both the childhood (Model 1) and adulthood (Model 2) adversity models. In the joint model (Model 3), where childhood and adulthood adversity were entered together, the total effect was only significant for adulthood adversity and not childhood adversity. Furthermore, in Models 1 (childhood adversity only) and 3 (childhood and adulthood adversity together), there were significant associations between childhood adversity and *better* executive functioning performance. In Models 2 (adulthood adversity only) and 3 (childhood adversity and adulthood adversity together), there were no associations between adulthood adversity and executive functioning. Across Models 1–3, there were no associations between executive functioning and internalizing symptoms. Consequently, there were no significant indirect effects of either childhood adversity or adulthood adversity on internalizing symptoms through executive functioning. Results for the childhood adversity, adulthood adversity, and joint childhood/adulthood adversity models are visualized in Figs. 1, 2 and 3 respectively.

**Table 3:** Total, direct, and indirect effects linking adversity to internalizing symptoms through executive functioning.

	Model 1: Childhood adversity as predictor		Model 2: Adulthood adversity as predictor		Model 3: Childhood/adulthood adversity as concurrent predictors	
	Unstandardized (standardized) effect	95% CI (unstandardized effect)	Unstandardized (standardized) effect	95% CI (unstandardized effect)	Unstandardized (standardized) effect	95% CI (unstandardized effect)
Total effect (*c* path)	0.44^***^ **(0.27)**	0.21, 0.69	0.57^***^ **(0.42)**	0.39, 0.75	0.15 (0.09)/0.57^***^ **(0.42)**	− 0.08, 0.40/0.39, 0.75
Direct effect (*c’* path)	0.44^***^ **(0.27)**	0.20, 0.70	0.57^***^ **(0.42)**	0.39 0.76	0.14 (0.09)/0.52^***^ **(0.39)**	− 0.11, 0.42/0.32, 0.74
Adversity → EF (*a* path)	0.04^*^ **(0.19)**	0.01, 0.07	0.00 (0.01)	− 0.03, 0.03	0.05^*^ **(0.23)**/− 0.01 (− 0.08)	0.01, 0.08/− 0.05, 0.01
EF→ Internalizing symptoms (*b* path)	0.05 (0.01)	− 1.54, 1.46	0.43 (0.05)	− 1.15, 1.75	0.29 (0.04)	− 1.28, 1.66
Indirect effect (a*b path)	0.00 (0.00)	− 0.06, 0.07	0.00 (0.00)	− 0.02, 0.03	0.01 (0.01)/− 0.00 (− 0.00)	− 0.06, 0.10/− 0.06, 0.02
Model Fit						
χ^2^ Statistic	20.35		18.89		20.80	
(df)	23		23		26	
p-value	0.62		0.71		0.75	
CFI	1.00		1.00		1.00	
TLI	1.00		1.00		1.00	
SRMR	0.03		0.03		0.03	
RMSEA	0.00		0.00		0.00	
(90% CI)	0.00, 0.05		0.00, 0.05		0.00, 0.04	
Close fit p-value	0.95		0.97		0.98	

95% CI–95% confidence interval.

EF–Executive function as assessed using the Cambridge Neuropsychological Test Automated Battery.

Note. Effects flagged at ***p ≤.001. **p ≤.01. *p ≤.05 reflect two-tailed *p*-value. Bolded coefficients are significant at *p* ≤.05 (two-tailed).

A similar pattern of findings was observed when replicating all models indexing total *severity* of stressors instead of total count (see Table S2 for model fit statistics and Figures S2–4 for model visualization). Finally, a non-pre-registered analysis in which adversity across the lifespan (0–24 years) was constructed by combining childhood and adulthood adversity revealed results that largely mirrored those of our adult and joint childhood/adulthood adversity models. Specifically, cumulative adversity scores (total count and severity) were significantly associated with internalizing symptoms, but no indirect effect through executive functioning was observed (see Table S3 for model fit statistics and Figures S5–S6 for model visualization).

### Stress sensitization model

Results of the stress-sensitization model investigating interactive effects of childhood adversity and adulthood adversity in predicting internalizing symptoms are reported in Table 4. A main effect of adulthood adversity, but not childhood adversity, on internalizing symptoms was detected. No significant interaction between childhood and adulthood adversity was observed. A similar pattern was observed when substituting adulthood adversity for recent-life adversity (see Table S4), and when examining *severity* of adversity instead of total counts (see Table S5 and Table S6). Thus, there was no evidence of stress sensitization in this study.

**Table 4:** Results of regression analyses with main and interaction effects of childhood adversity and adulthood adversity on internalizing symptoms.

	Model 1: Childhood Adversity as Predictor	
	Unstandardized (Standardized) effect	95% CI (unstandardized effect)
Main effect childhood adversity	0.17 (0.10)	− 0.05, 0.42
Main effect adulthood adversity	0.53^***^ **(0.39)**	0.32, 0.75
Childhood adversity*adulthood adversity interaction	− 0.01 (− 0.03)	− 0.04, 0.03

^***^p ≤.001. ^**^p ≤.01. ^*^p ≤.05.

Note. Analyses control for the same covariates noted above in the primary models.

## Discussion

Associations between experiences of adversity and internalizing symptoms are well documented, but the specific processes through which adversity is associated with internalizing symptoms, as well as potential variations in findings based on timing of adversity exposure, are underexplored. Emerging adulthood is a period of heightened risk for internalizing symptoms, making it an ideal stage in which to explore transdiagnostic processes associated with psychopathology risk^4^. One such transdiagnostic process is challenges with executive functioning^16,19,24,25,49–51^, and while it is broadly understood that childhood adversity is negatively associated with executive functioning in children, comparatively less is known about associations between adulthood adversity and executive functioning during emerging adulthood. Additionally, no research to date has investigated how associations between both childhood and adulthood adversity, considered together, relate to executive functioning and its associations with internalizing symptoms during emerging adulthood.

Results of the present study support prior research linking adversity exposure and internalizing symptoms^52,53^. In separate models, childhood and adulthood adversity were both significantly associated with internalizing symptoms in this community sample of emerging adults. However, when childhood and adulthood adversity were entered into a joint model as competing predictors, only associations between adulthood adversity and internalizing symptoms remained significant. Notably, childhood and adulthood adversity were only moderately correlated, suggesting there is some shared underlying vulnerability to adversity over time, but that experiences across these periods are not isomorphic. Together, these results suggest that adulthood adversity is more strongly related to internalizing symptoms than childhood adversity, which was not what we expected in our pre-registered hypotheses (i.e., that childhood adversity would be more strongly associated with internalizing symptoms). Our original hypotheses were largely grounded in the robust literature suggesting that childhood represents a developmentally sensitive period characterized by heightened neuroplasticity and sensitivity to environmental input^38^. However, we acknowledge that there are alternative approaches beyond sensitive period models. In one of the only studies directly comparing different lifespan models, both *recency* and *accumulation* models outperformed *sensitive period* models in predicting child psychopathology symptoms^54^. Indeed, while not what we had originally hypothesized, our results align with such recency models, suggesting that psychopathology outcomes are most strongly linked to more proximal experiences of adversity^54,55^. These findings add to a growing literature suggesting that adversity during adulthood may be just as important as that which occurs during childhood and adolescence in predicting mental health symptoms during early adulthood^17,18^. This may be due to increased vulnerability to adversity during emerging adulthood as a function of substantial social and neurobiological change that occurs during this period^56^. However, we cannot rule out that the stronger association between adulthood adversity (in comparison to childhood adversity) and internalizing symptoms is a statistical artifact that reflects the timing of measurement—specifically, participants in this cross-sectional study may have been able to more reliably report on experiences of adulthood adversity given their proximity to data collection, whereas retrospective experiences of childhood adversity may have been biased, unreliable, or otherwise impacted by their current experiences. Indeed, controlling for adulthood adversity (proximal experience) reduced the effect of childhood adversity (past experience) on internalizing symptoms to non-significance. We cannot know whether elimination of the childhood adversity effect upon inclusion of adulthood adversity reflects such a bias, or whether it may be reflective of some other process such as *stress proliferation* or *stress generation*^57,58^. If correct, this suggests that part of the effect of childhood adversity on internalizing symptoms is due to its catalyzing effect on later stress exposure proximal to psychopathology. Future longitudinal work with prospective measurement is required to further test these possibilities. It is also important to note that both childhood and adulthood adversity, as well as internalizing symptoms, were indexed using self-report, leading to risk of shared method bias and possible inflation of the strength of associations between constructs.

Our hypotheses related to the indirect effects of adversity on internalizing symptoms through executive functioning were not supported. Across all models, executive functioning was not associated with internalizing symptoms, and no indirect effects were observed. However, although adulthood adversity was not associated with executive functioning, childhood adversity was associated with *better* executive functioning performance in both the childhood adversity-only and joint childhood/adulthood adversity models. This finding was counter to our pre-registered hypotheses, but is consistent with the *Hidden Talents* perspective, which theorizes that individuals who have experienced harsh or unpredictable childhood environments may exhibit adaptations in specific facets of cognition, memory, or learning to meet the demands of threatening and unpredictable environments^37^. Importantly, however, the results of the current study do not constitute a direct test of the Hidden Talents framework, with three primary challenges to this interpretation: (i) childhood adversity was indexed across a range of experiences rather than specifically querying threatening or unpredictable experiences; (ii) executive functioning was indexed as a broad construct inclusive of several individual abilities, not all of which may show similar adaptations to adversity; and (iii) the sample comprised predominantly University students, a select group who may be able to adapt to early adversity and maintain higher executive function that is directly tied to their likelihood of entering higher education in the first place. Accordingly, we offer the Hidden Talents framework as a cautious interpretive framework rather than direct confirmatory evidence of this perspective.

Finally, exploratory analyses investigating adulthood adversity as a moderator of associations between childhood adversity and internalizing symptoms vis-à-vis a stress-sensitization model were not supported. Additional exploratory analyses substituting adulthood adversity for recent-life adversity, or investigating severity instead of total count of exposures, also did not reveal any stress-sensitization effects. Thus, while the results may be consistent with a theory of stress proliferation as noted above, they do not suggest that childhood adversity lowers the threshold for tolerating future stress, or that future stress amplifies the effects of childhood adversity on internalizing symptoms (i.e., stress sensitization).

In general, results of the present study are consistent with recent research emphasizing the importance of investigating developmental timing and severity of adversity as factors which may increase explanatory power in characterizing impacts on health and well-being^59–61^, while also emphasizing the importance of implementing a life-course approach to adversity measurement^62^. Although our results revealed variation based on retrospective reports of timing of exposures related to childhood and adulthood adversity, no notable differences were detected when indexing total counts of exposures relative to severity of exposures. This pattern of findings may suggest that timing of exposure is an important factor in characterizing how experiences of adversity impact development during emerging adulthood.

### Strengths and limitations

A noteworthy strength of the present study is the diversity of assessment methods, ranging from use of a structured adversity inventory, self-report of internalizing symptoms, and objective measurement of executive functioning across a number of performance-based metrics. The use of performance-based metrics of executive functioning in adversity research with emerging adults is rare^28^, and this approach reduces the risk of shared method bias that many studies relying solely on participant reports cannot overcome. Moreover, use of the STRAIN enabled us to explore factors such as timing and severity of adversity, which permits a deeper understanding of adversity characteristics in relation to outcomes. Finally, our sample was racially and ethnically diverse, with more than two-thirds of the sample identifying as non-White – European/North American. Although the subgroups were not large enough to permit stratified analyses, this sample diversity increases confidence in the generalizability of the results.

Several limitations are also noteworthy. First, while use of the CANTAB is considered a significant strength overall, we did not include a measure of inhibitory control to go along with our measurement of attention, cognitive flexibility, planning/problem-solving, and working memory. Task selection was limited based on the use of the online version of the CANTAB, which did not have a compatible online version of a response inhibition task at the time of data collection. Second, we chose to focus on internalizing problems given that these are highly prevalent during emerging adulthood, but greater attention to externalizing problems is warranted given these are also consistently associated with both childhood adversity^63^ and executive function^64^. Indeed, contrasting associations of childhood and adulthood adversity with internalizing and externalizing problems, including the shared and distinct indirect pathways through which these effects operate, is a rich area for future research. Current meta-analytic work shows that retrospective accounts of adversity are more strongly associated with adult internalizing symptoms relative to externalizing symptoms^65^. In not controlling externalizing symptoms, we cannot know whether observed associations are specific to internalizing problems or instead apply more widely across the psychopathology spectrum. Third, it should be noted that data collection overlapped with the COVID-19 pandemic. This may have heightened experiences of proximal stressors (i.e., adulthood adversity) and increased risk of psychopathology, thus inflating associations between adulthood adversity and internalizing symptoms. This may be viewed as either a limitation or a substantively important set of findings reflecting heightened risk during a period of developmental vulnerability (i.e., emerging adulthood) nested within a period of contextual vulnerability (i.e., the pandemic). As we did not measure COVID-specific stressors in this study, we cannot ascertain how this risk specifically may have impacted the results. Relatedly, since data collection took place during the pandemic, another possible limitation is that participation was fully online and unsupervised. While data quality and attention checks suggested these factors were minimally impactful, we cannot know for certain how they may have impacted the results. Additionally, some findings may have been influenced by selection bias, with the sample largely comprised of university students who may have been higher functioning in the first place and resolved with respect to their adversity experiences. These individuals may have had the cognitive, interpersonal, or material resources that allowed them to overcome or benefit from adversity in a way that most others could not. Still, the positive association between childhood adversity and executive function did not extend to internalizing symptoms, with higher levels of adversity linked to higher internalizing symptoms in this sample. These “tradeoffs” following adversity—such as the benefits afforded by enhanced executive function skills but at the cost of greater psychopathology risk—are aligned with the Hidden Talents perspective^37^. This raises questions about the circumstances under which these tradeoffs occur, and whether such effects are, in part, driven by sampling factors. An additional limitation is the fact that our measurement of childhood adversity was not sufficient to test specific developmental epochs of adversity exposure during childhood and adolescence. It should also be noted that our focus on adult life adversity may introduce an additional source of recency bias given that exposures are more proximal and may thus be more reliably recalled than experiences of childhood adversity. Furthermore, the STRAIN does not allow us to ascertain distinct versus overlapping types of adversity endorsed during childhood and adulthood. Thus, we cannot rule out that differences in the types or intensity of stressors across periods also contribute to observed effects, rather than the timing of common stressors per se. Finally, as a cross-sectional study, we cannot make any inferences regarding causality or directionality of effects, and participant reports may have been subject to recency or other reporting biases, as noted above. Still, it is worth noting that retrospective accounts of childhood adversity demonstrate stronger associations with psychopathology than objectively- or prospectively-ascertained childhood adversity^66,67^. Although retrospective reports frequently show stronger associations with psychopathology, we view both prospective and retrospective assessments of early-life adversity as providing distinct and complementary insights, and we encourage the use of both approaches in future research. While participant self-reports of adversity are therefore justified, the study design does not permit causal conclusions regarding the nature of effects.

## Conclusion

This study emphasizes the importance of investigating associations between adversity at different periods of development in relation to internalizing symptoms in emerging adulthood. Specifically, results highlight adulthood adversity as demonstrating a stronger association with internalizing symptoms during emerging adulthood relative to childhood adversity. Although executive functioning did not account for associations between either childhood or adulthood adversity with internalizing symptoms, results showed that childhood adversity was associated with increased executive functioning task performance. These results raise the possibility of alterations in cognition that reflect adaptation to early stress, though these appear independent of (i.e., do not relate to) mental health risk during emerging adulthood.

## Supplementary Information

Below is the link to the electronic supplementary material.

Supplementary Material 1

## Data Availability

Data for this study are not available for public use because this was not retained as part of the participant consent process prior to study commencement. Code and output files for all analyses are available upon reasonable request.
