# Supplementary material for: Lifetime stressor exposure, executive functioning, and internalizing symptoms during emerging adulthood
Source: Sci Rep. 2026 Mar 31;16:15593. doi: 10.1038/s41598-026-44738-4 (PMC13187190; doi:10.1038/s41598-026-44738-4)

| **Table S1. Expanded bivariate associations between study variables** | 16. IED (Rescaled) |  |  |  |  |  |  |  |  |  |  |  |  |  |  |  | ^-^ | ^***^*p* ≤.001. ^**^*p* ≤ .01. ^*^*p* ≤ .05 reflect two-tailed *p*-value. Bolded coefficients are significant at *p* < .05 (two-tailed). |
| --- | --- | --- | --- | --- | --- | --- | --- | --- | --- | --- | --- | --- | --- | --- | --- | --- | --- | --- |
|  | 15. RVPA |  |  |  |  |  |  |  |  |  |  |  |  |  |  | - | **.35***** |  |
|  | 14. OTS |  |  |  |  |  |  |  |  |  |  |  |  |  | - | **.53***** | **.38***** |  |
|  | 13. SWM (Rescaled) |  |  |  |  |  |  |  |  |  |  |  |  | - | .**32***** | **.42***** | **.28***** |  |
|  | 12. Recent-Life Adversity Severity |  |  |  |  |  |  |  |  |  |  |  | **-** | -.13 | -.05 | -.02 | -.08 |  |
|  | 11. Adulthood Adversity Severity |  |  |  |  |  |  |  |  |  |  | **-** | **.85***** | -.03 | .01 | .07 | -.05 |  |
|  | 10. Childhood Adversity Severity |  |  |  |  |  |  |  |  |  | **-** | **.33***** | **.47***** | -.04 | .11 | .11 | .04 |  |
|  | 9. Recent-Life Adversity |  |  |  |  |  |  |  |  | - | **.44***** | **.79***** | **.96***** | **-.16*** | -.07 | -.08 | -.08 |  |
|  | 8. Adulthood Adversity |  |  |  |  |  |  |  | - | **.78***** | **.49***** | **.91***** | **.81***** | -.06 | .04 | .06 | -.03 |  |
|  | 7. Childhood Adversity |  |  |  |  |  |  | - | **.43***** | .41*** | **.92***** | **.28***** | .4**1***** | -.07 | .13 | .13 | .05 |  |
|  | 6. Internalizing Symptoms (K10) |  |  |  |  |  | - | **.30***** | **.42***** | **.44***** | **.32***** | **.44***** | **.49***** | .00 | .07 | .04 | -.08 |  |
|  | 5. Monthly Household Income (Reference Category: $10,000+ CAD) |  |  |  |  | - | -.04 | -.06 | -.07 | -.01 | -.09 | -.03 | -.02 | .01 | .02 | -.05 | .06 |  |
|  | 4. Years of Education |  |  |  | - | .07 | **-.14*** | **-.16*** | -.03 | **-.21**** | **-.16*** | .03 | **-.17*** | **.15*** | **.19**** | **.29***** | .11 |  |
|  | 3. Gender (Reference Category: Women & Transwoman) |  |  | - | **.18*** | -.06 | **-.16*** | -.12 | -.00 | -.06 | **-.15*** | -.01 | -.05 | .01 | -.02 | .01 | .01 |  |
|  | 2. Ethnicity (Reference Category: White – European/ North American) |  | - | -.02 | -.11 | .00 | -.09 | **-.15*** | -.10 | **-.15*** | -.13 | -.09 | -.13 | -.06 | **-.17*** | **-.16*** | -.04 |  |
|  | 1. Age | - | -.02 | **.25***** | **.61***** | .09 | -.12 | **-.26***** | .02 | **-.15*** | **-.26***** | .09 | -.11 | **.24***** | **.15*** | **.23**** | .14 |  |
|  |  | 1. | 2. | 3. | 4. | 5. | 6. | 7. | 8. | 9. | 10. | 11. | 12. | 13. | 14. | 15. | 16. |  |

| **Table S2:** Total, direct, and indirect effects linking adversity severity to internalizing symptoms through executive functioning | | | | | | | | |
| --- | --- | --- | --- | --- | --- | --- | --- | --- |
|  | Model 1: Childhood adversity severity as predictor | |  | Model 2: Adulthood adversity severity as predictor | |  | Model 3: Childhood/adulthood adversity severity as simultaneous predictors | |
|  | *Unstandardized*  *(standardized) effect* | 95% CI (unstandardized effect) |  | *Unstandardized*  *(standardized) effect* | 95% CI (unstandardized effect) |  | *Unstandardized*  *(standardized) effect* | 95% CI (unstandardized effect) |
| Total effect (*c* path) | **.18^***^ (.28)** | .08, .27 |  | **.25^***^ (.44)** | .18, .33 |  | **.**09 (.14)/ **.25^***^ (.44)** | -.01, .18/ .18, .33 |
| Direct effect (*c’* path) | **.18^***^ (.28)** | .08, .27 |  | **.25^***^ (.44)** | .18, .33 |  | .08 (.13)/ **.23^***^ (.40)** | -.02, .18/ .15, .31 |
| Adversity severity → EF (*a* path) | **.01^*^ (.16)** | .00, .03 |  | .00 (-.00) | -.01, .01 |  | **.02^*^ (.19)**/ -.01 (-.07) | .00, .03/ -.02, .01 |
| EF→ Internalizing symptoms (*b* path) | .10 (.01) | -1.51, 1.48 |  | .49 (.06) | -1.06, 1.80 |  | .32 (.04) | -1.23, 1.62 |
| Indirect effect (*a*b* path) | .00 (.00) | -.02, .03 |  | .00 (.00) | -.01, .01 |  | .01 (.01)/ -.00 (-.00) | -.02, .04/ -.02, .01 |
| Model Fit |  |  |  |  |  |  |  |  |
| χ^2^ Statistic | 20.12 | |  | 18.95 | |  | 21.39 | |
| (df) | 23 | |  | 23 | |  | 26 | |
| p-value | .63 | |  | .70 | |  | .72 | |
| CFI | 1.00 | |  | 1.00 | |  | 1.00 | |
| TLI | 1.00 | |  | 1.00 | |  | 1.00 | |
| SRMR | .03 | |  | .03 | |  | .03 | |
| RMSEA | .00 | |  | .00 | |  | .00 | |
| (90% CI) | .00, .05 | |  | .00, .05 | |  | .00, .04 | |
| Close fit p-value | .95 | |  | .97 | |  | .98 | |
| 95% CI–95% confidence interval.  EF–Executive function as assessed using the Cambridge Neuropsychological Test Automated Battery.  *Note.* Effects flagged at ^***^*p* ≤ .001. ^**^*p* ≤ .01. ^*^*p* ≤ .05 reflect two-tailed *p*-value. Bolded coefficients are significant at *p* < .05 (two-tailed). | | | | | | | | |

| **Table S3:** Total, direct, and indirect effects linking cumulative adversity total counts and severity to internalizing symptoms through executive functioning | | | | | | |
| --- | --- | --- | --- | --- | --- | --- |
|  | Model 1: Cumulative adversity total count as predictor | |  | Model 2: Cumulative adversity severity as predictor | |  |
|  | *Unstandardized*  *(standardized) effect* | 95% CI (unstandardized effect) |  | *Unstandardized*  *(standardized) effect* | 95% CI (unstandardized effect) |  |
| Total effect (*c* path) | **.35^***^ (.42)** | .25, .45 |  | **.16^***^ (.44)** | .11, .20 |  |
| Direct effect (*c’* path) | **.35^***^ (.42)** | .25, .46 |  | **.16^***^ (.44)** | .11, .20 |  |
| Adversity severity → EF (*a* path) | .01 (.09) | -.01, .03 |  | .00 (.08) | -.00, .01 |  |
| EF→ Internalizing symptoms (*b* path) | .13 (.02) | -1.38, 1.40 |  | .16 (.02) | -1.33, 1.43 |  |
| Indirect effect (*a*b* path) | .00 (.00) | -.02, .03 |  | .00 (.00) | -.01, .01 |  |
| Model Fit |  |  |  |  |  |  |
| χ^2^ Statistic | 19.73 | |  | 19.71 | |  |
| (df) | 23 | |  | 23 | |  |
| p-value | .66 | |  | .66 | |  |
| CFI | 1.00 | |  | 1.00 | |  |
| TLI | 1.00 | |  | 1.00 | |  |
| SRMR | .03 | |  | .03 | |  |
| RMSEA | .00 | |  | .00 | |  |
| (90% CI) | .00, .05 | |  | .00, .05 | |  |
| Close fit p-value | .96 | |  | .96 | |  |
| 95% CI–95% confidence interval.  EF–Executive function as assessed using the Cambridge Neuropsychological Test Automated Battery.  *Note.* Effects flagged at ^***^*p* ≤ .001. ^**^*p* ≤ .01. ^*^*p* ≤ .05 reflect two-tailed *p*-value. Bolded coefficients are significant at *p* < .05 (two-tailed). | | | | | |  |

| **Table S4:** Results of exploratory regression analyses with main and interaction effects of childhood and recent-life adversity on internalizing symptoms | | | |  |
| --- | --- | --- | --- | --- |
|  | Model 1: Childhood adversity as predictor | |  |  |
|  | *Unstandardized*  *(standardized) effect* | 95% CI (unstandardized effect) |  |  |
| Main effect childhood adversity | .18 (.11) | -.02, .42 |  |  |
| Main effect recent-life adversity | **1.19**^***^ **(.37)** | .70, 1.69 |  |  |
| Childhood adversity*recent-life adversity interaction | .03 (.05) | -.06, .12 |  |  |
| ^***^*p* ≤ .001. ^**^*p* ≤ .01. ^*^*p* ≤ .05.  *Note.* Analyses control for the same covariates noted above in the primary models. | | | |  |

| **Table S5:** Results of exploratory regression analyses with main and interaction effects of childhood and adulthood adversity severity on internalizing symptoms | | | |
| --- | --- | --- | --- |
|  | Model 1: Childhood adversity severity as predictor | |  |
|  | *Unstandardized*  *(standardized) effect* | 95% CI (unstandardized effect) |  |
| Main effect childhood adversity severity | .07 (.12) | -.01, .17 |  |
| Main effect adulthood adversity severity | **.22**^***^ **(.38)** | .14, .30 |  |
| Childhood adversity severity*adulthood adversity severity interaction | .00 (.06) | -.00, .01 |  |
| ^***^*p* ≤ .001. ^**^*p* ≤ .01. ^*^*p* ≤ .05.  *Note.* Analyses control for the same covariates noted above in the primary models. | | | |

| **Table S6:** Results of exploratory regression analyses with main and interaction effects of childhood and recent-life adversity severity on internalizing symptoms | | | |
| --- | --- | --- | --- |
|  | Model 1: Childhood adversity severity as predictor | |  |
|  | *Unstandardized*  *(standardized) effect* | 95% CI (unstandardized effect) |  |
| Main effect childhood adversity severity | .03 (.05) | -.06, .12 |  |
| Main effect recent-life adversity severity | **.33**^***^ **(.41)** | .21, .47 |  |
| Childhood adversity severity*recent-life adversity severity interaction | .00 (.09) | -.00, .01 |  |
| ^***^*p* ≤ .001. ^**^*p* ≤ .01. ^*^*p* ≤ .05.  *Note.* Analyses control for the same covariates noted above in the primary models. | | | |


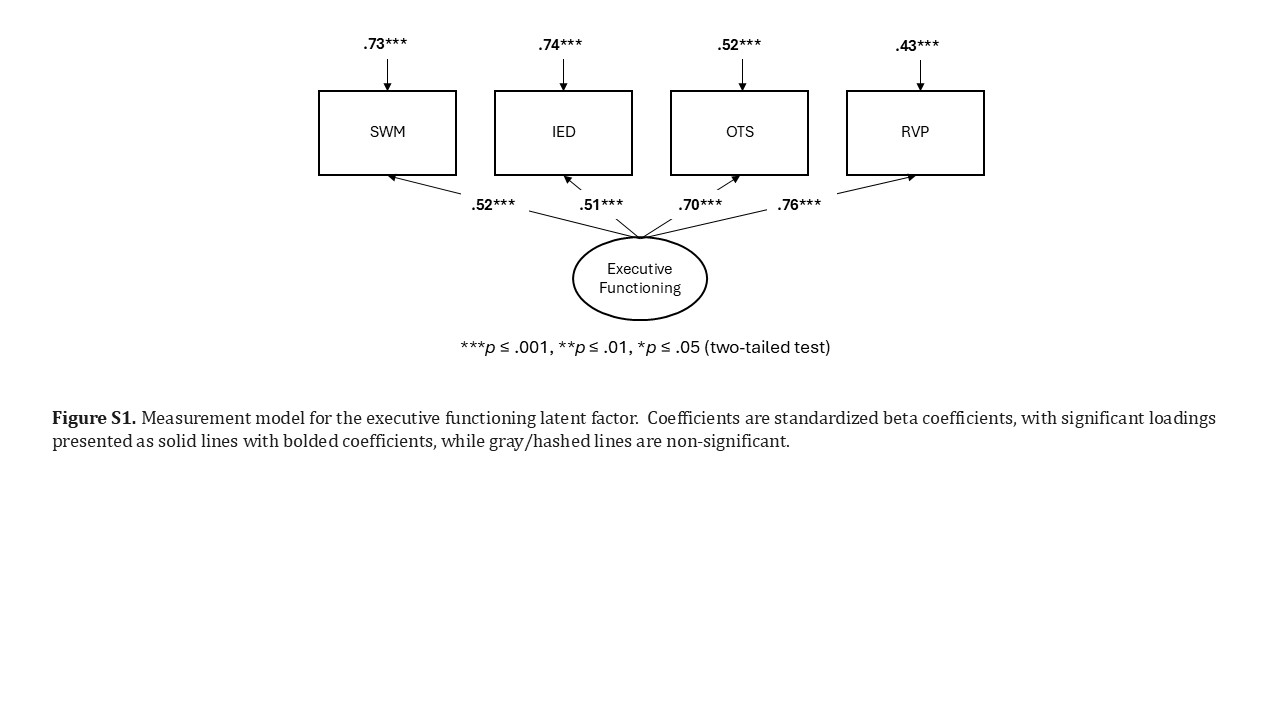


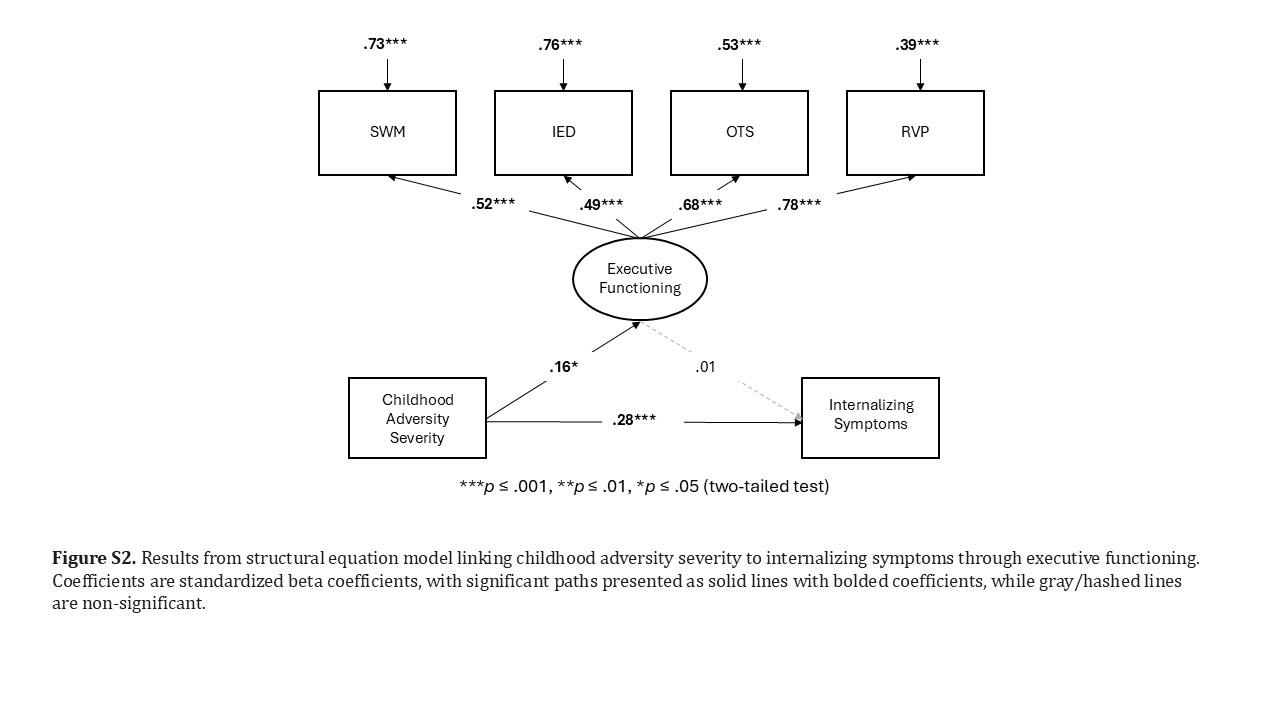


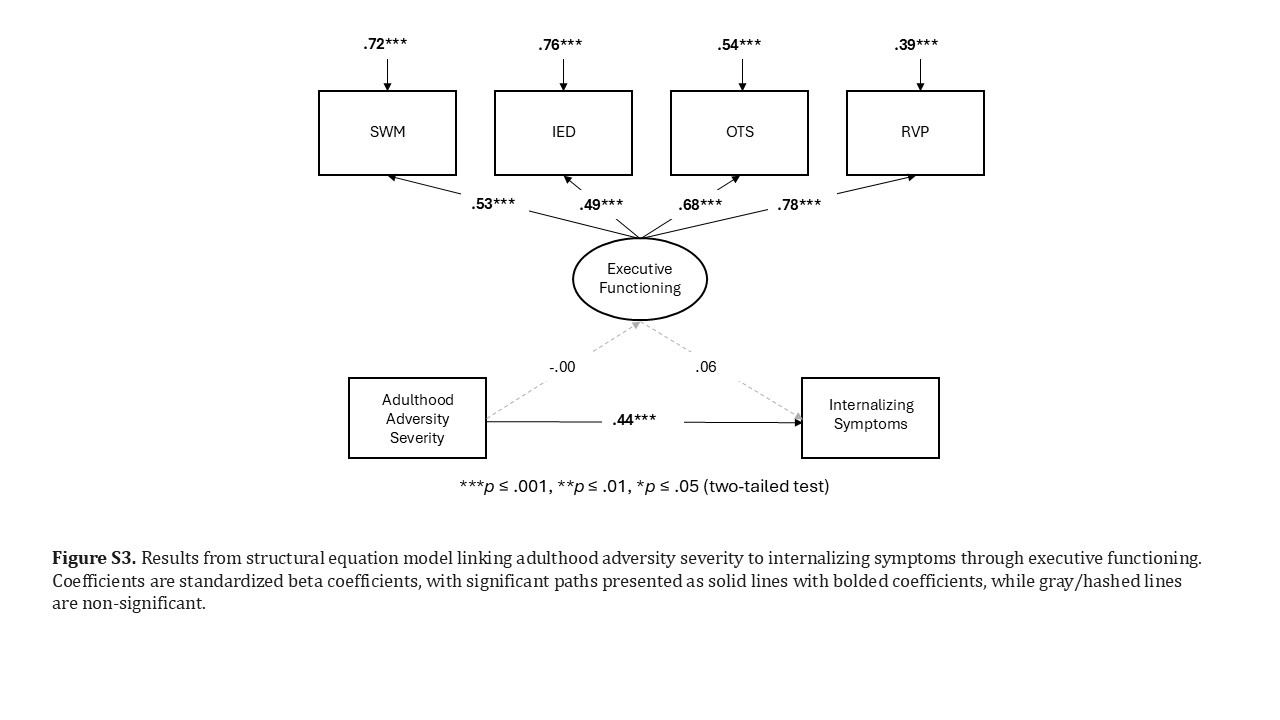


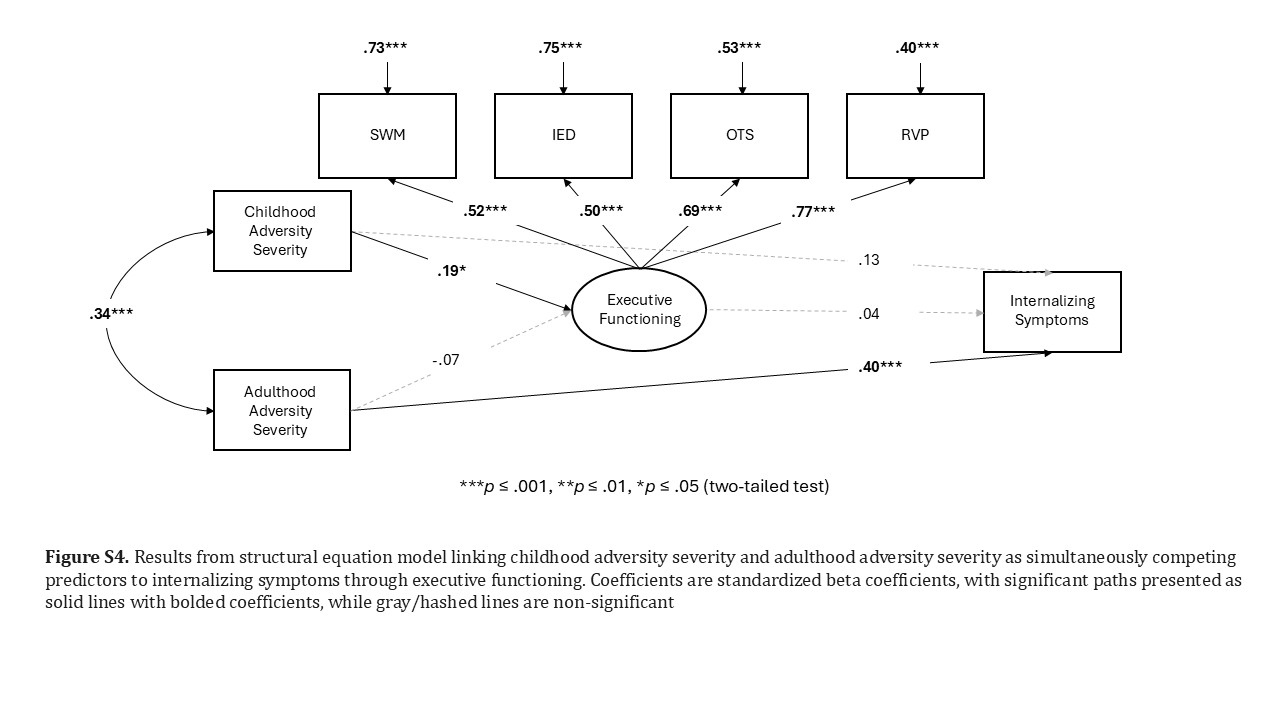


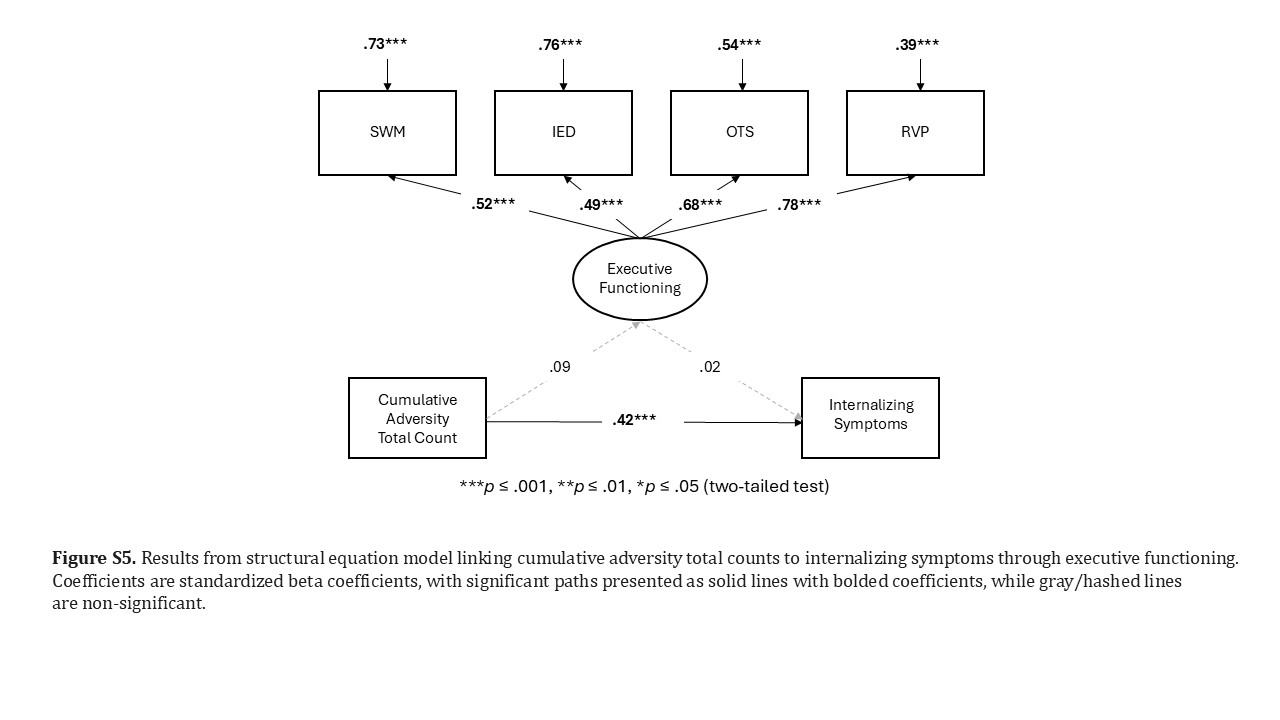


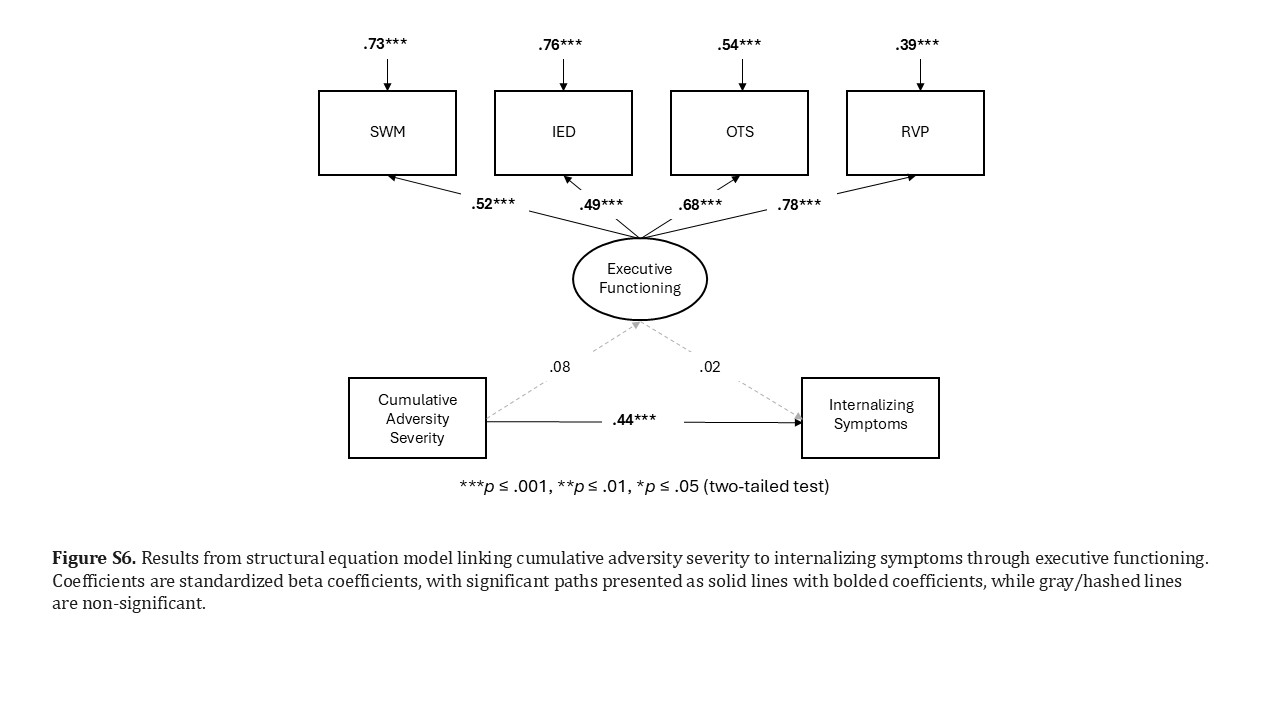

Supplement: Supplementary file 1 — Supplementary Material 1 [file 41598_2026_44738_MOESM1_ESM.docx]
